# Prevalence of hypertension among Patients Seeking Care in selected health facilities in the Southern Province of Sierra Leone

**DOI:** 10.1371/journal.pgph.0003281

**Published:** 2025-04-29

**Authors:** Samuel Maxwell Tom Williams, Sahr Foday, Richard Wadsworth, Ibrahim K. Foday, Esther Marie Williams, George Mayeh Fefegula, Mohamed S. P. Koker

**Affiliations:** 1 Department of Biological Sciences, Njala University, Freetown, Sierra Leone; 2 Njala University Hospital, Njala University, Freetown, Sierra Leone; 3 Ministry of Agriculture, Forestry and Food Security, Livestock Division, Freetown, Sierra Leone; PLOS: Public Library of Science, UNITED STATES OF AMERICA

## Abstract

Hypertension is a multifactorial disease caused by various environmental, lifestyle, and genetic factors. Hypertension is a major contributor to cardiovascular mortality in Sierra Leone, with the prevalence estimated to be 29.4% among males and 31.6% among females. The study aimed to determine the prevalence of high blood pressure among people seeking medical treatment at four health facilities in the southern province of Sierra Leone.We obtained anonymized individual records of blood pressure measurements from four health facilities (Njala University Hospital, Dandabu CHC, Futa Pejeh CHC, and Njala University Teaching Health Center). A total of 1,793 outpatient records were collected. Linear regression was used with age (years) and sex as independent variables. The total prevalence of hypertension in our study was 36.8%. The average male patient was an adult (37.5 years) with healthy blood pressure (123/75.4 mm/Hg). The average female patient was relatively young (27.6 years) with healthy blood pressure (113.8/72.8 mm/Hg). Age and sex significantly affect the increase of blood pressure in the study. Based on this finding, we recommend the improvement of healthcare infrastructure and affordable antihypertensive medication for all patients.

## Introduction

Non-communicable diseases (NCDs) are increasing in West Africa, with high blood pressure being a major risk factor for cardiovascular disease [1–3]. Hypertension is responsible for 54% of strokes and 47% of ischemic heart attacks [4]. Hypertension is a multifactorial disease caused by various environmental and genetic factors. Lifestyle risk factors such as smoking, drinking alcohol, eating a diet high in fats, sugars, and salts, being overweight, and not getting enough physical activity are strongly linked to hypertension as aging are significant risk factors for hypertension [5]. Even a small change in blood pressure, of only a few millimeters of mercury (mm), can have serious consequences [6]. As well as the direct problems caused by hypertension it has secondary implications for diseases such as cancer, cardiovascular diseases, and diabetes [7]. Previous studies have shown that not only does hypertension possibly promote incidental cancer risk, but prehypertension (SBP: 120–139 mmHg or DBP: 80–89 mmHg) and antihypertension treatment may also promote increased risk of incidental cancer [8,9]. These diseases, increasingly common in Sub-Saharan Africa, must be managed alongside hypertension [10]. Diagnosis and treatment for hypertension are often unavailable or under-available in low-income populations [11]. Even though early identification and treatment of hypertension effectively saves lives and reduces costs to families and health systems, the problem is often ignored.

According to the World Health Organization, hypertension is the leading cause of death in Africa, with a prevalence of 29.4% among males and 31.6% among females [12]. Other studies have also shown a similar alarming prevalence of hypertension in the country; it is well-known that hypertension increase with age. This relationship between age and blood pressure explains some of the differences in reported percentage for the adult population as a whole; for example, a recent national survey found that 22% of people aged 18 + years have hypertension [13], while a study conducted in the Bo District found that 49% of people aged 40 + years have hypertension [14].

A study comparing Sierra Leone and the Gambia found a prevalence of 46.2% among women and 43.2% among men, with a total prevalence of 44.8% [15]. Furthermore, Lebbie et al. [16] found a prevalence of 12.0% hypertension in the undergraduate student population at Njala University.

Hence, identifying the prevalence of hypertension in Sierra Leone and other countries is a first step toward developing and prioritizing policies and practices to reduce the morbidity and mortality associated with high blood pressure. The main objective of this study is to determine the prevalence of high blood pressure (HBP) using the American College of Cardiology/American Heart Association 2017 guidelines among people seeking medical treatment at selected health facilities.

## Methodology

### Study area

The study was conducted at four health facilities; Njala University Hospital (NUH), Dandabu Community Health Post (CHP), Futa Pejeh Community Health Center (CHC), and Njala University Teaching Health Center (NUTHC). The NUH is situated on the main campus of Kori chiefdom Moyamba district (approximately 8°06’49.0“N 12°04’26.6” W). Dandabu CHP is located at Kpanda- Kabondeh chiefdom of Pujehun district (approximately 7°28’39.6”N 11°41’06.8” W), Futa Pejeh CHC is also situated in Futa Pejeh chiefdom of Pujehun district (approximately 7°33’38.6”N 11°34’27.2” W). The NUTHC is situated in the Kakua chiefdom of Bo district (approximately 7°57’00.1”N 11°44’54.7” W). These facilities are surrounded by towns and villages where people seek medical treatment before referral to provide a service that falls outside the professional competence of the CHCs and CHPs.

The health facilities were strategically chosen for several reasons. Firstly, each facility serves as a primary healthcare provider and hotspot for communities within a 25–50-kilometer radius from the nearest government hospital, making them critical points of access for individuals in these rural and peri-urban areas. Additionally, these facilities are known for their relatively high patient flow, which enables the collection of substantial data for assessing hypertension prevalence among a diverse demographic.

### Targeted participants

The targeted participants for this study were outpatients aged ≥15 years who attended medical treatment for any reason. Selection criteria for these facilities included their capacity to provide outpatient services and record routine vital measurements, including blood pressure. These health centers have consistent data collection processes. They are located in regions with limited healthcare access, allowing the study to capture data representative of underserved populations in the southern province.

#### Inclusion and exclusion criteria.

The inclusion criteria for this study were individuals aged 15 years and older who visited the selected health facilities and had their blood pressure recorded. While blood pressure was not measured for every patient at these facilities, this study only includes data from individuals who had their blood pressure recorded during their visit. To ensure data quality and relevance, only records with complete blood pressure information were analyzed, allowing us to estimate hypertension prevalence among patients attending these health centers accurately.

While national surveys often measure blood pressure in populations aged 18 and older, emerging research indicates that hypertension can begin as early as adolescence. Studies have shown that adolescents, particularly those with risk factors such as obesity, poor diet, and sedentary lifestyles, are increasingly susceptible to elevated blood pressure and early-onset hypertension [17,18]. Including patients aged 15 and above thus enables us to capture early hypertension prevalence in younger individuals, contributing to a more comprehensive understanding of hypertension trends in Sierra Leone’s population.

### Data collection and study design

Secondary data was collected in the research, focusing on people who visited the selected health facilities to seek medical treatments. In cases where multiple blood pressure readings were recorded for a patient, this study used the mean (average) of all recorded readings to represent the patient’s blood pressure. Taking an average of multiple BP readings of the same patients provides a more accurate assessment by minimizing potential variability due to stress, time of day, or measurement conditions. For consistency, BP measurement used in this study were recorded at the time of patient registration. This approach enhances the reliability of our prevalence data by ensuring that measurements are taken under comparable conditions across facilities.

The data ranges for the covers different periods for each facility;

- Dandabu CHP (2020–2021),
- NUH (2021–2022),
- NUTHC Center (2017–2022) and
- Futa Pejeh CHC (2016–2022).

To provide a comprehensive prevalence estimate, all data were merged into a single dataset and analyzed collectively while accounting for facility, age, and sex variations. Given the different timeframes, our prevalence estimates reflect the overall burden of hypertension across the cumulative study period rather than a specific calendar year. We acknowledge that this approach may introduce variability across facilities due to temporal differences, which is considered a study limitation.

Some of the social characteristics from the data collected from the patients’ logbook were age, gender, and record of blood pressure. However, there was no record for height and weight (calculating body mass index, was not available) on the patient’s log book.

### Ethical considerations

This study exclusively utilized secondary data that had already been collected by the participating health centers in line with established best practices. Ethical clearance was obtained from the Directorate of Research and Development at Njala University (Approval Reference: 2022/ea/01). Given the retrospective nature of the study, written consent was not directly obtained from individual patients. Instead, institutional consent was sought from each health facility, with formal permissions granted to access and use anonymized data for research purposes.

#### Anonymization process.

To ensure strict confidentiality, data abstraction procedures were designed to eliminate all personally identifiable information. Beyond excluding names and phone numbers, any other unique identifiers, such as patient IDs or addresses, were omitted from the dataset. This approach ensured that the data remained entirely anonymous and untraceable to individual patients, upholding ethical standards for handling sensitive health information in retrospective studies.

### Sample size

A total of 1,793 outpatient records were collected; Futa Pejeh CHC 840 patients, NUTHC 61, NUH 717, and Dandabu CHP 175 patients. Extrapolating from the sample to the general population is difficult for three reasons; (i) we are restricted to those seeking health care in a formal setting, and so does not include those making use of traditional medicine for similar conditions (ii) the age distribution of our sample does not mirror that age distribution of the general population of the study area.

### Statistical analysis

Using the blood pressure (BP) classification recommended in 2017 by the American College of Cardiology (ACC)/American Heart Association (AHA) hypertension [19] and World Health Organization [20], blood pressure was categorized into:

- Low: less than 90/60
- Normal: Less than 120/80 mm Hg;
- Prehypertension: Systolic between 120–129 and diastolic less than 80;
- Stage 1: Systolic between 130–139 or diastolic between 80–89;
- Stage 2: Systolic at least ≥ 140 or diastolic at least ≥ 90 mm Hg;
- Hypertensive crisis: Systolic over 180 and diastolic over 120, with patients needing prompt changes in medication if there are no other indications of problems or immediate hospitalization if there are signs of organ damage.

The Prevalence of hypertension in the form of normal, low, elevated, and Stage-1 and stage-2 Hypertension were all determined. The impact of age, and sex, as predictor variables on systolic blood pressure (SBP) and diastolic blood pressure (DBP) were tested using simple linear regression. Using the simple linear regression model, we estimated the magnitude of change in the outcome variables and then expressed our estimate along with a 95% confidence interval based on that estimate. When the probability level was less than 0.05, it was considered significant. SPSS 28.0 software (SPSS, Inc.) and Microsoft Excel were used for the statistical analysis.

## Result

Using a specific prevalence technique in the study analyses, the study revealed a 36.8% prevalence of hypertension among the study population, categorically, 22.1% elevated blood pressure, 6.0% having stage-1 hypertension, and 8.6% having stage-2 hypertensive cases (Table 1).

**Table 1:** Hypertension Prevalence by Study Site and Blood Pressure Category.

Row Labels	(n)	Normal	Low	Elevated	Stage-1	Stage-2	Sum of HBP
		n (%)	n (%)	n (%)	n (%)	n (%)	n(%)
Sum of individuals	1793	1103 (61.5)	31 (1.7)	397 (22.1)	107 (6.0)	155 (8.6)	659 (36.8)
Study site							
Dandabu CHP	175	82 (46.9)	1 (0.6)	14 (8.0)	11 (6.3)	67 (38.3)	92 (52.6)
Futa Pejeh CHC	840	630 (75.0)	13 (1.5)	186 (22.1)	8 (1.0)	3 (0.4)	197 (23.5)
NUTHC	61	33 (54.1)	1 (1.6)	10 (16.4)	10 (16.4)	7 (11.5)	27 (44.3)
NUH	717	358 (49.9)	16 (2.2)	187 (26.1)	78 (10.9)	78 (10.9)	343 (47.8)
Age range (years)							
15-24	728	511 (70.2)	19 (2.6)	153 (21.0)	24 (3.3)	21 (2.9)	198 (27.2)
25-34	750	483 (64.4)	10 (1.3)	183 (24.4)	35 (4.7)	39 (5.2)	257 (34.3)
35-44	119	57 (47.9)	1 (0.8)	29 (24.4)	14 (11.8)	18 (15.1)	61 (51.3)
45-54	82	23 (28.0)		15 (18.3)	12 (14.6)	32 (39.0)	59 (72.0)
55-64	50	13 (26.0)		6 (12.0)	14 (28.0)	17 (34.0)	37 (74.0)
65+	64	16 (25.0)	1 (1.6)	11 (17.2)	8 (12.5)	28 (43.8)	47 (73.4)
Sex							
Male	303	120 (39.6)	6 (2.0)	84 (27.7)	45 (14.9)	48 (15.8)	177 (58.4)
Female	1490	983 (66.0)	25 (1.7)	313 (21.0)	62 (4.2)	107 (7.2)	482 (32.3)

**Fig 1:**
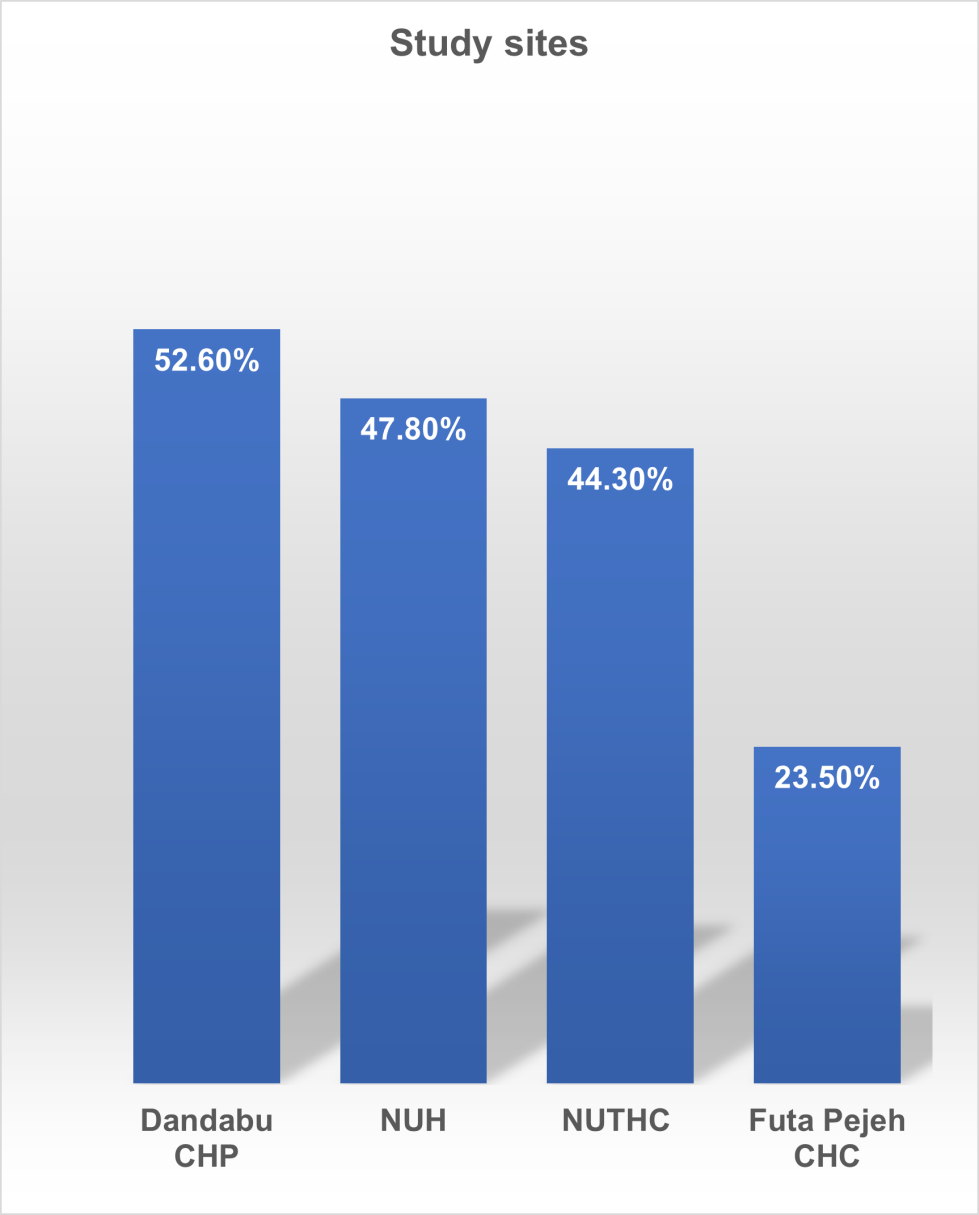
Site-Specific Hypertension Prevalence.

With regards to location, Dandabu CHP had the highest prevalence (52.6%) followed by, NUH with 47.8%, NUTHC with 44.3%, and Futa Pejeh CHC with 1.4%. The prevalence of hypertension increases as the age increases in the study population (Table 1; Fig 1).

**Fig 2:**
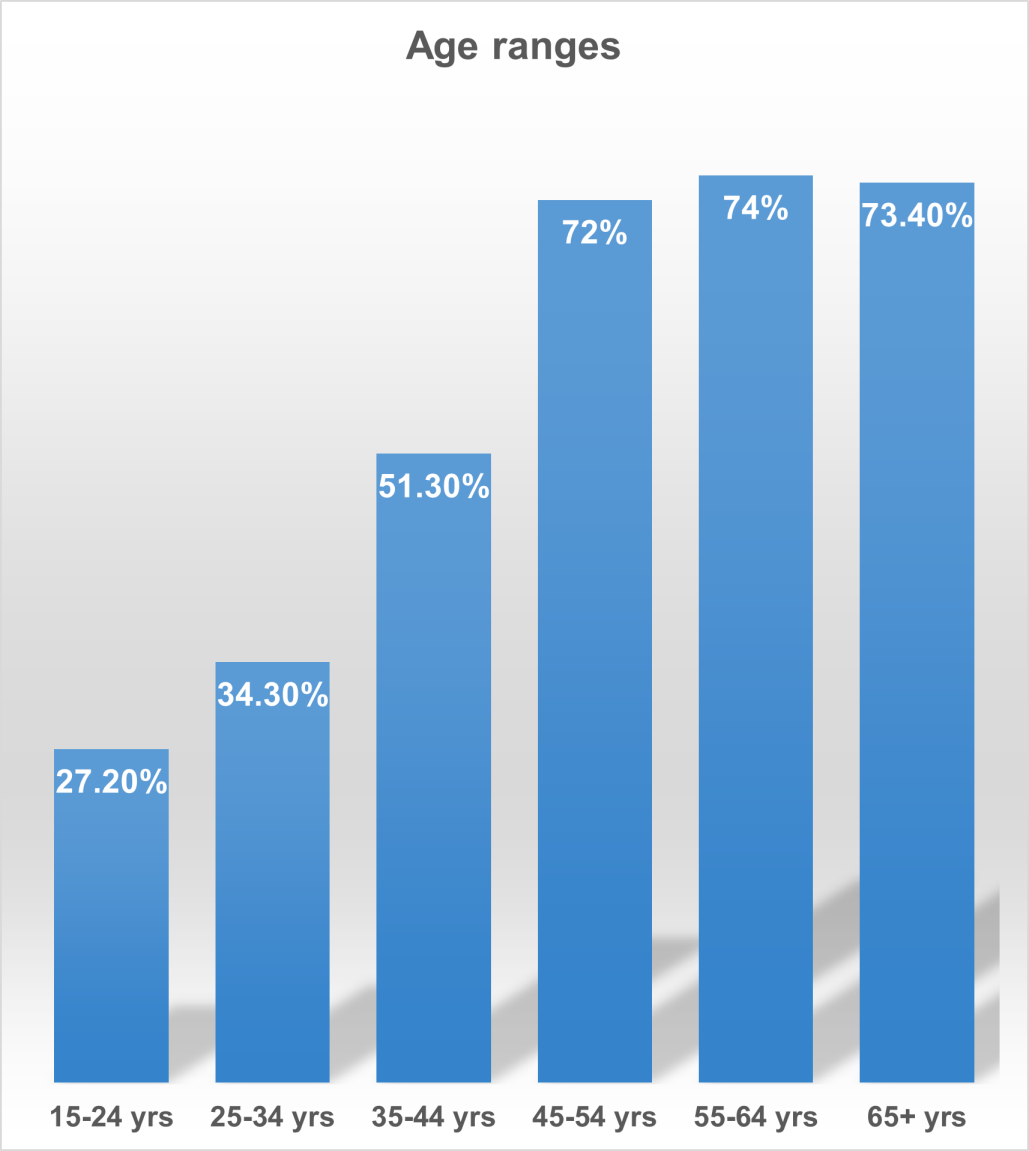
Age-Specific Hypertension Prevalence.

The highest prevalence rate was among the age group 55–64 years with 74.0% prevalence and 65 + years with 73.4% with prevalence, followed by 45–54 years 72.0% prevalence, 35–44 years with 51.3% prevalence, 25–34 years with 34.3% prevalence and the lowest prevalence among the youngest age group (15–24 years) with 27.2% prevalence (Fig 2; Table 1).

**Fig 3:**
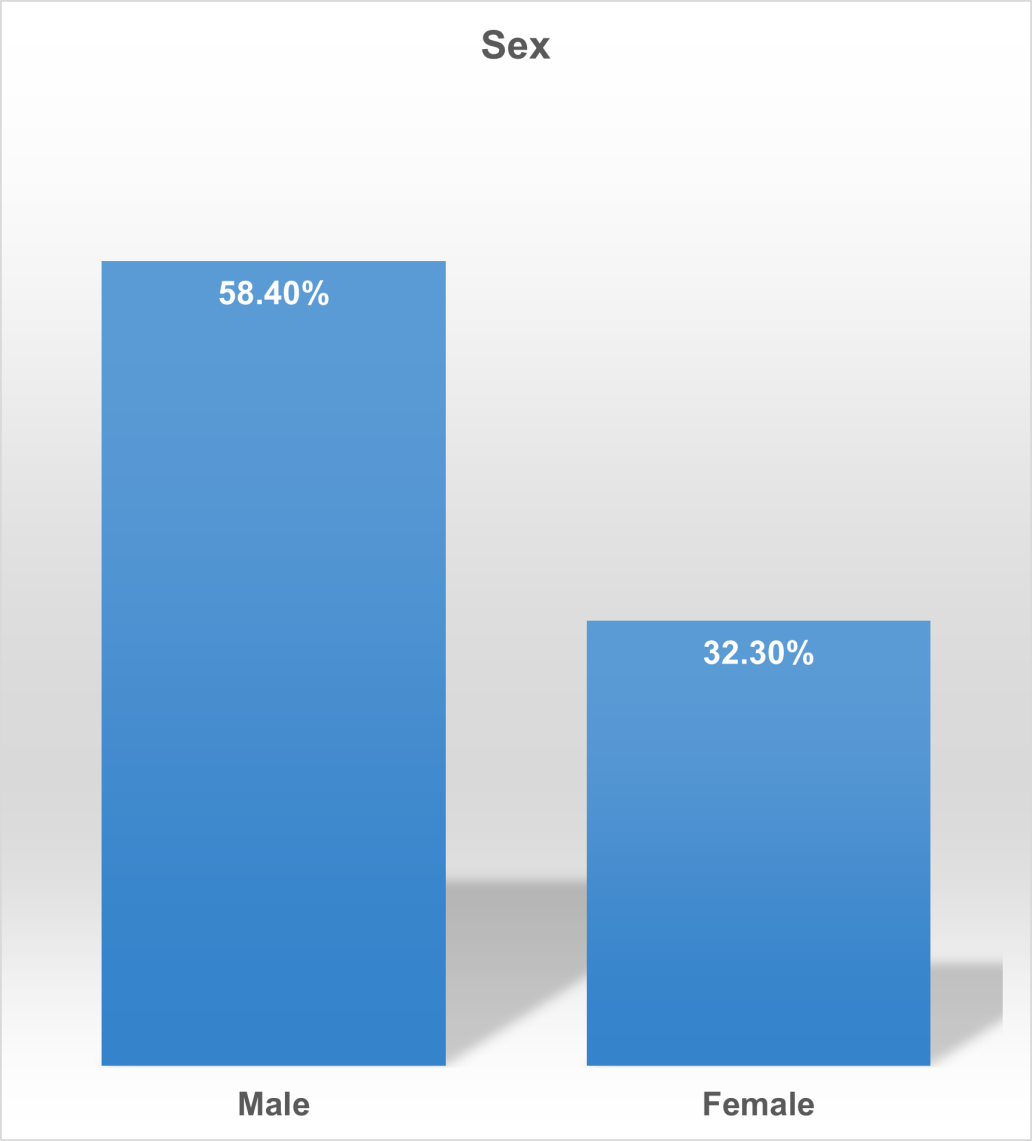
Gender-Specific Hypertension Prevalence.

The study also revealed that there is sexual dimorphism in hypertension prevalence such that men accounted for 58.4% male-specific prevalence as compared to women with 32.3% female-specific prevalence (Fig 3; Table 1)

Table 2 shows that the average male was an adult (37.5 years) with a prehypertensive condition (123 mmHg and 75.4 mmHg SBP and DBP). Also, the average female was a young lady in her mid to late twenties (27.6 years) with prehypertension (113.8 mmHg and 72.8 mmHg SBP and DBP respectively).

**Table 2:** Average Age and Blood Pressure (SBP & DBP) by Gender.

Variables	Males	Females
Average age (years)	37.5	27.6
Average Systolic (mmHg)	123	113.8
Average Diastolic (mmHg)	75.4	72.8

Table 3 shows that age has a significant positive effect on systolic blood pressure, while sex does not have a significant impact according to the data presented. Based on the description, it seems that age has a significant correlation with both systolic and diastolic blood pressure. This means as age increases, so does blood pressure, which is a common finding in medical studies. Because the coefficient is zero and or negative (-3.008 for SBD; and 0.081 for DBP), it suggests that males, on average, have higher systolic blood pressure than females. However, the description indicates that the sex variable is not statistically significant (p-value = 0.137 for SBP; and 0.081 for DBP), which means there’s no strong evidence of a difference in systolic blood pressure between females and males in this particular sample

**Table 3:** Regression Analysis of Blood Pressure Predictors.

Labels	Systolic blood pressure		Diastolic blood pressure	
	Coefficient	P-value (95% CI)	Coefficient	P-value (95% CI)
Age (in years)	0.628	<0.001 (0.513 ± 0.744)	0.126	<0.001 (0.081 ± 0.171)
Sex	-3.008	0.137 (-6.969 ± 0,954)	-1.367	0.081 (-2.903 ± 0.168)

## Discussion

In this study, we investigated the prevalence of HBP among people attending the selected health facilities of the southern province of Sierra Leone using the American College of Cardiology/American Heart Association 2017 guidelines.

The total prevalence of hypertension in our study was 36.8% which is higher than a similar study (12.0%) conducted at Njala University of Moyamba district among randomly selected undergraduate students [16]. The prevalence found in our study is also higher than the reported prevalence rate of 22% from a hospital-based study conducted in Freetown [13], and much lower than (the 49.0%) elicited in Bo district 49% [14].

The study revealed a significantly higher prevalence of hypertension among males compared to females, a finding consistent with trends observed in other regions. Sexual dimorphism is evident in the prevalence of hypertension [21]. This study indicated a higher specific prevalence of hypertension among males (58.4%) compared to females (32.3%). The disparity in hypertension prevalence between men and women is substantial; for example, the Heart Disease and Stroke Statistics 2021 update noted that the age-adjusted prevalence of hypertension in the US from 2015 to 2018 for those over the age of 20 was 51.7% for males and 42.8% for females [21]. Research suggests that biological, behavioral, and social factors all contribute to this disparity. For instance, men are generally at a higher risk of developing hypertension earlier in life, possibly due to differences in hormone levels, particularly the protective role of estrogen in women before menopause [22]. After menopause, however, the prevalence in women often increases, sometimes surpassing that in men, as estrogen levels decline [23].

Lifestyle and behavioral factors may further contribute to these differences. Studies indicate that men are more likely to engage in behaviors associated with hypertension risk, such as higher alcohol consumption, smoking, and dietary patterns high in sodium and fat. Societal factors, such as higher levels of occupational stress in men, might also play a role [24]. In addition, men are less likely to seek preventive healthcare services compared to women, potentially leading to undiagnosed and untreated hypertension in earlier stages. This delay in healthcare-seeking behavior can result in higher prevalence and severity when they receive a diagnosis.

The notably high prevalence of hypertension at Dandabu Community Health Post (CHP) could be attributed to several contextual factors. Dandabu CHP serves a primarily rural population with limited access to healthcare services, which may contribute to undiagnosed and untreated hypertension. This facility is also one of the primary health centers within a 25–50 Kilometer radius, suggesting that individuals who seek care here may present with more advanced or poorly managed conditions. Socioeconomic factors, such as high poverty rates and limited access to health education, could further exacerbate the risk of hypertension in this community. Additionally, lifestyle factors, including dietary habits, physical inactivity, and stress related to economic hardship, may contribute to elevated blood pressure levels in the population served by Dandabu CHP.

This high prevalence is a serious cause for worry since the participants were healthy persons unaware of their blood pressure status. Several previous reports are linking certain lifestyles and traits, such as high cholesterol, smoking, and diabetes to HBP and the reliability of using carotid thickening as an indicator of impending blood pressure and cardiovascular problems [25–27] but our study does not, however, cover those parameters.

However, one of the underlying causes of the increase in hypertension among the study population is that most of the people within the study population hardly come to the clinic for medical diagnosis. Instead, they rely on family members and friends to influence their decisions. A study on community understanding of NCD and healthcare-seeking behavior in Sierra Leone observed that participants generally approached healthcare services when they experienced symptoms, hoping for a quick cure and permanent solution [28], also people who seek advice from family and friends about which medical help should be sought, possibly resulting in seeking care from informal and traditional healers, using the formal healthcare system as a last resort [28] possibly in part due to the financial vulnerability.

### Potential causes of high hypertension prevalence in the study population

The high prevalence of hypertension observed in this study may stem from a combination of genetic predispositions, lifestyle factors, and limited access to healthcare. In Sierra Leone and other low- and middle-income countries, socioeconomic constraints can contribute significantly to hypertension risk. Financial insecurity often limits access to quality healthcare, preventive services, and education on healthy lifestyles, resulting in higher rates of undiagnosed and untreated hypertension [29].

Dietary habits may also play a key role. High salt intake, along with diets low in fresh fruits and vegetables, has been linked to hypertension in multiple studies [30] (WHO, 2013). Economic barriers often limit food choices, pushing individuals toward cheaper, processed foods high in salt and unhealthy fats. Additionally, low levels of physical activity, partly due to urbanization and sedentary lifestyles, can contribute to higher blood pressure levels [31].

Psychosocial stressors are another factor, as individuals in the study area may experience economic hardship, job insecurity, and social instability all linked to elevated blood pressure. Chronic stress can increase blood pressure over time by elevating stress hormones, which can lead to blood vessel constriction and increased heart rate [32]. Furthermore, healthcare infrastructure challenges in Sierra Leone mean that preventive care and early diagnosis of hypertension are limited, especially in rural areas. Many individuals only seek medical attention when symptoms become severe, often resulting in the detection of hypertension at more advanced stages [33,34]. Together, these factors underscore the complex interplay between lifestyle, socioeconomic status, and healthcare access in driving hypertension prevalence in the population.

## Conclusion

Most studies on hypertension in West Africa (including this one) are relatively small (a few hundred participants) and concerned only with patients attending a health clinic or hospital. What is needed is a nationwide random survey to ensure that all sectors of the population are covered. Our study revealed an alarming prevalence of hypertension (36.8%) among patients visiting community health centers. Age was a predictor of increased blood pressure (both SBP & DBP) but not gender.

To improve hypertension management in underserved areas, community health facilities should be equipped with reliable, easy-to-use blood pressure monitors to enable regular screenings. Specialized training for healthcare providers at all levels, especially in rural centers, is essential to ensure consistent application of hypertension diagnosis and management guidelines. Establishing mobile health clinics would further extend preventive screenings, education, and basic treatment to remote regions, bridging access gaps for those unable to visit fixed healthcare facilities. In addition, partnerships with pharmaceutical suppliers and government programs can ensure affordable access to antihypertensive medications, improving adherence among low-income patients.

In conclusion, implementing health communication campaigns and taxes on unhealthy foods could have a transformative impact on hypertension prevention and control in Sierra Leone. Health communication campaigns are effective in raising awareness about hypertension risks and promoting healthier lifestyles [35]. For example, Finland’s “North Karelia Project” used media and community education to reduce cardiovascular risk, leading to significant declines in hypertension and related mortality [36]. Similarly, campaigns in the U.K. and Australia have encouraged dietary improvements and physical activity, resulting in healthier population outcomes [37]. Taxes on unhealthy foods, particularly those high in salt, sugar, and saturated fats, have also shown measurable success; Mexico’s tax on sugary beverages led to a 7.6% reduction in consumption within two years, especially among lower-income households who face higher NCD risks [38]. Hungary’s “public health product tax” on high-sugar and high-salt foods spurred manufacturers to reformulate products, reducing population exposure to these harmful ingredients [39]. Adopting similar strategies in Sierra Leone could help reduce hypertension rates, create a healthier food environment, and increase public awareness, offering a feasible approach to address the country’s hypertension burden.

## Supporting information

S1 Data Raw data file of patient records used in the analysis. (XLSX)

S1 Text Full version of the research manuscript titled *“Prevalence of hypertension among Patients Seeking Care in selected health facilities in the Southern Province of Sierra Leone”.* Included as supporting information for reference. (DOCX)
